# Supplementary material for: Benzothiazole Derivatives as Dual Modulators of PGE2 and GABAergic Signaling in Skeletal Muscle
Source: bioRxiv. 2026 Jun 3:2026.05.30.728982. Preprint. [Version 1] doi: 10.64898/2026.05.30.728982 (PMC13252022; doi:10.64898/2026.05.30.728982)
Supplement: Supplement 1 [file media-1.pdf]

Supplementary Materials for

Benzothiazole Derivatives as Dual Modulators of PGE2 and GABAergic Signaling in Skeletal Muscle

Marian N. Aziz *et al.*

*Corresponding authors. Email: lovely@chemistry.msstate.edu; marco.brotto@uta.edu

This file includes:

Figs. S-Figure 1

Tables: S-Table 1

Spectra: **Data S3 to S14**

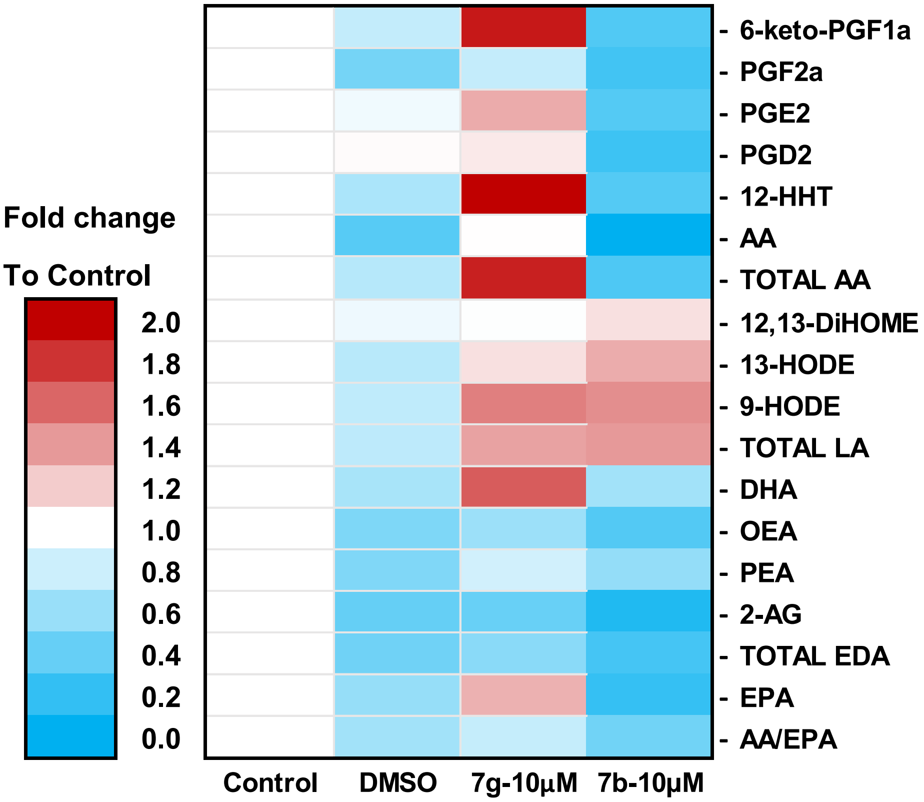

S-Figure 1: Benzothiazole agents 7b and 7g regulate the lipid profile in C2C12 skeletal muscle cells. Heatmap of quantified LMs normalized to positive control indicate that benzothiazole agent **7b** significantly decrease the lipid profile while agent **7g** increase the LMs concentration compared to other groups.

Supplementary materials Table 1

	2-D docking poses	Binding affinity	Interactions Details				
			Ligand	Receptor	Interactions	Distance	E (kcal/mol)
1	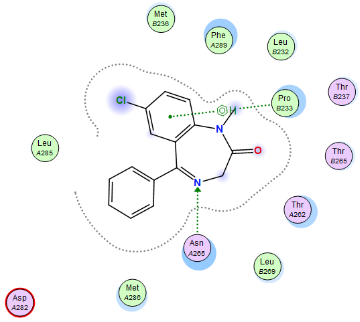	-6.03813934	N16	ASN 265	H-acceptor	2.84	-2.1
			6-ring	PRO 233	pi-H	3.75	-0.6
2	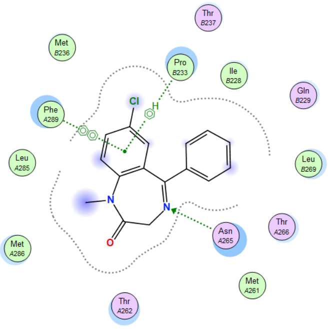	-6.42177391	6-ring	PRO 233	pi-H	4.14	-0.5
			6-ring	PHE 289	pi-pi	3.77	-0.0
3	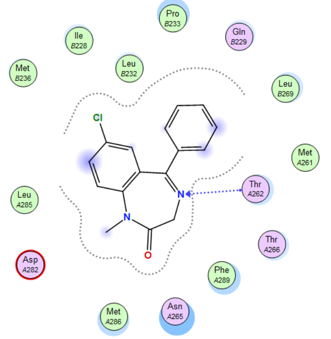	-6.33674431	N16	THR 262	H-acceptor	3.49	-0.6
4	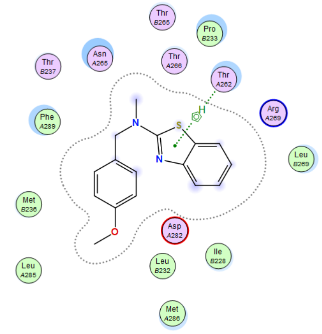	-6.9812212	5-ring	THR 262	pi-H	3.64	-0.5

**5**	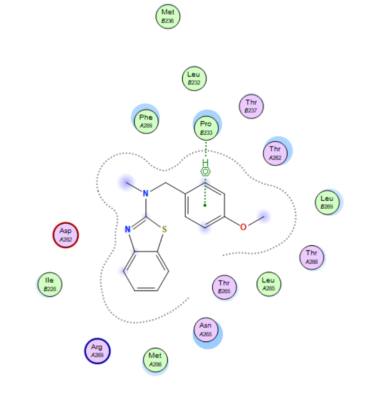	-6.74971676	6-ring	PRO 233	pi-H	3.77	-0.6
**6**	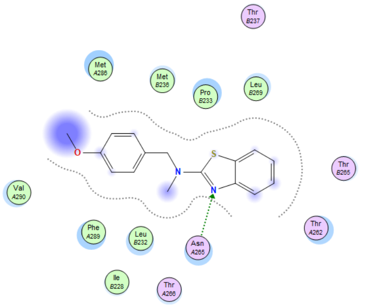	-6.36131859	N17	ASN 265	H-acceptor	3.23	-0.5
**7**	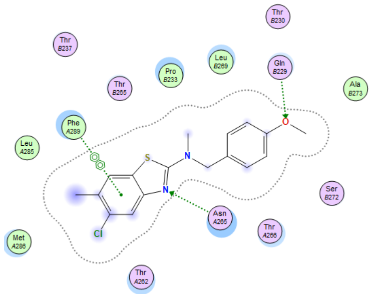	-6.66467094	N17	ASN 265	H-acceptor	3.47	-0.8
			O31	GLN 229	H-acceptor	2.81	-0.7
			6-ring	6-ring/PHE 289	pi-pi	3.91	-0.0
**8**	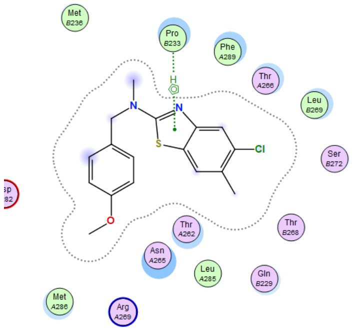	-6.72870636	5-ring	PRO 233	pi-H	3.75	-0.6

**9**	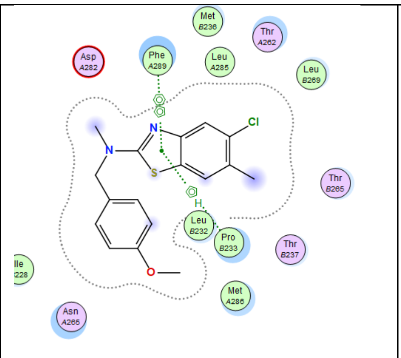	-6.72259903	5-ring	PRO 233	pi-H	3.82	-0.8
			5-ring	6-ring/PHE 289	pi-pi	3.98	-0.0
**10**	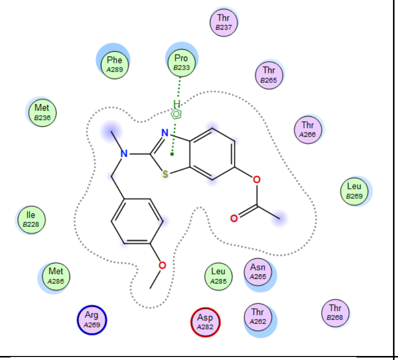	-7.71505594	5-ring	PRO 233	pi-H	3.73	-0.7
**11**	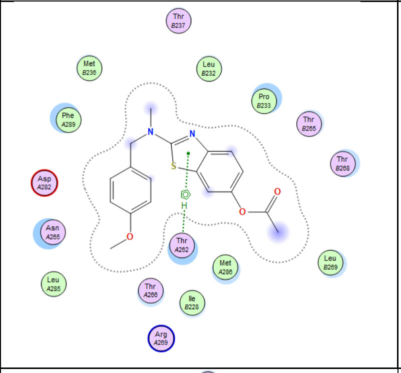	-7.6685605	5-ring	THR 262	pi-H	3.96	-0.5
**12**	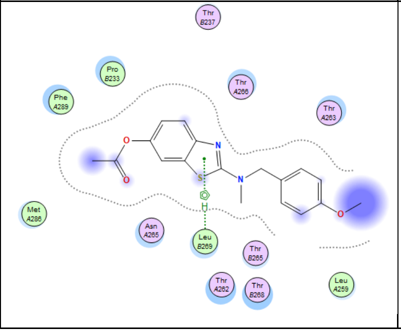	-7.6685605	5-ring	LEU 269	pi-H	3.88	-0.5

**13**	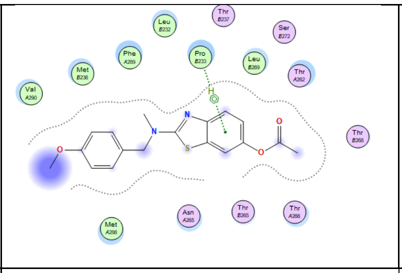	-7.01082182	6-ring	PRO 233	pi-H	4.17	-0.5
**14**	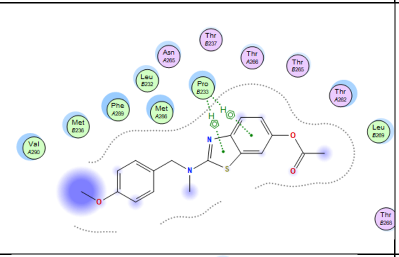	-6.92033482	6-ring	PRO 233	pi-H	3.74	-0.5
			5-ring	PRO 233	pi-H	4.05	-0.6
**15**	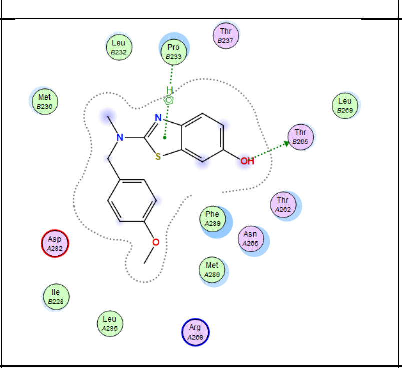	-6.86683846	O27	THR 265	H-donor	3.07	-0.7
			5-ring	PRO 233	pi-H	3.74	-0.9
**16**	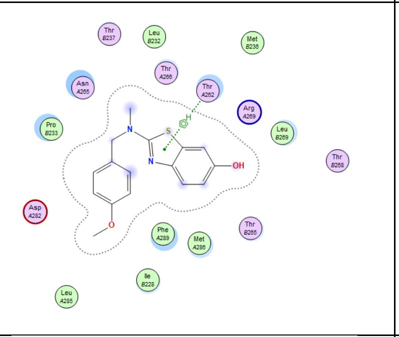	-6.97476816	5-ring	THR 262	pi-H	3.61	-0.5
**18**	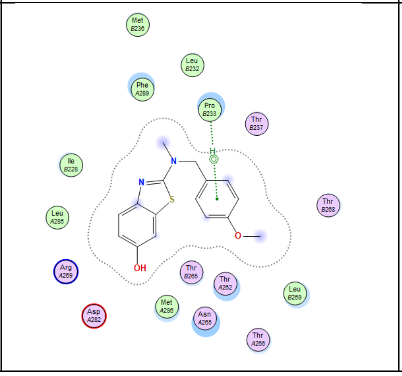	-6.77980042	6-ring	PRO 233	pi-H	4.08	-0.7

Supplementary materials Data S3-S14: ^{1}H and ^{13}C NMR spectra for:

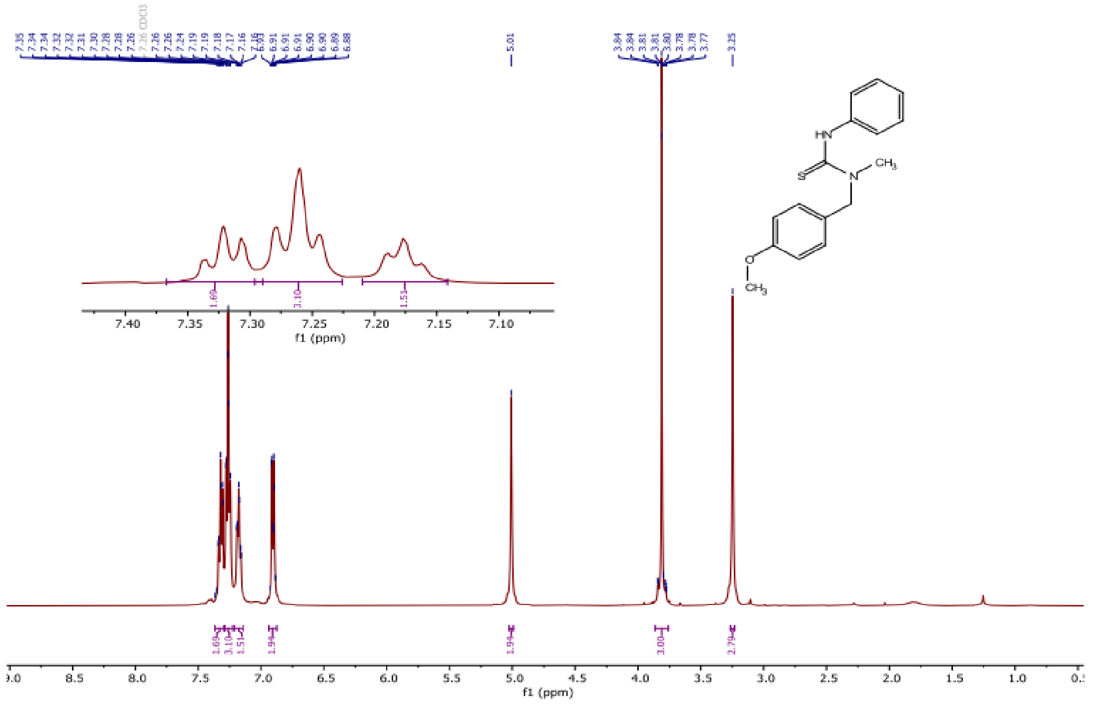

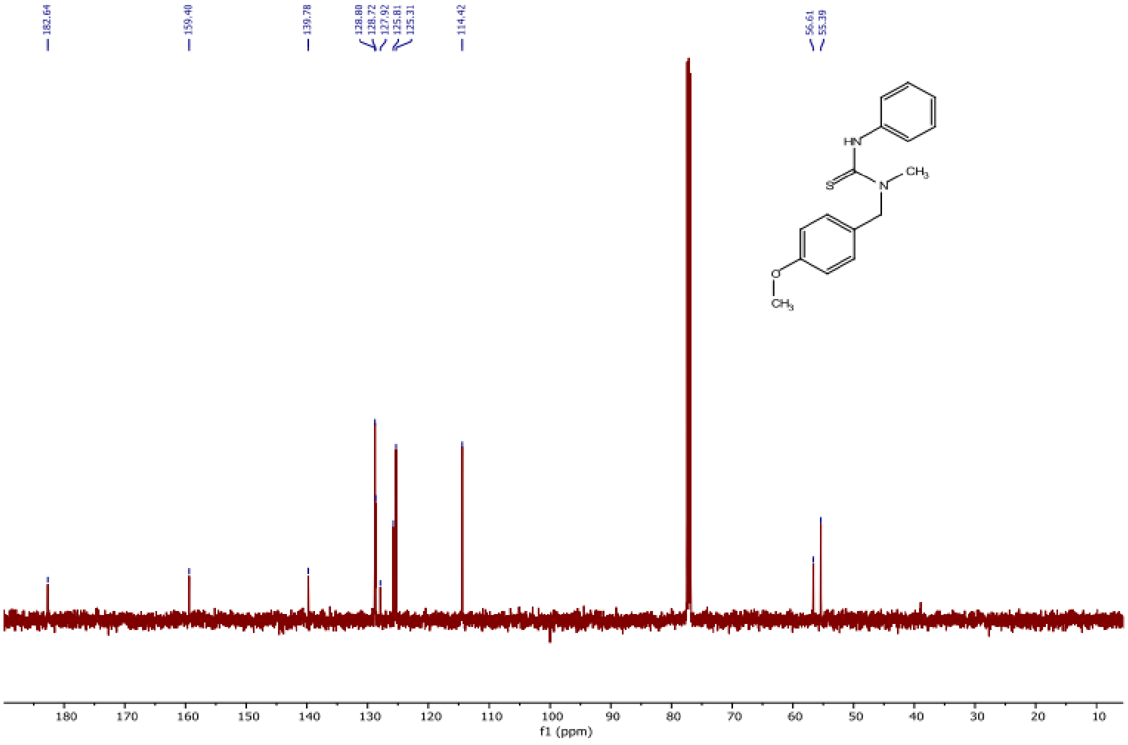

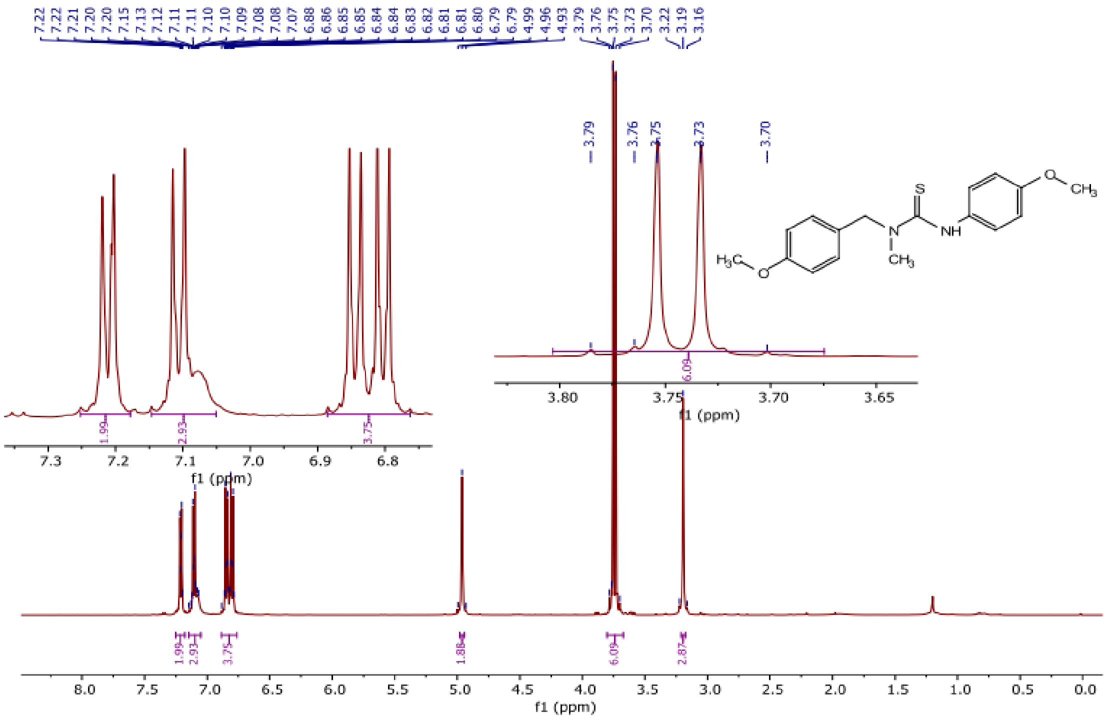

7.22 7.22 7.21 7.20 7.20 7.15 7.13 7.12 7.11 7.11 7.10 7.10 7.09 7.08 7.08 7.07 6.88 6.86 6.85 6.85 6.84 6.84 6.83 6.82 6.81 6.81 6.80 6.79 6.79 4.99 4.96 4.93 3.79 3.76 3.75 3.73 3.70 3.22 3.19 3.16
3.79 3.76 3.75 3.73 3.70
6.09
3.80 3.75 3.70 3.65
f1 (ppm)
1.99 2.93 3.75
7.3 7.2 7.1 7.0 6.9 6.8
f1 (ppm)
1.99 2.93 3.75 1.88 6.09 2.87
8.0 7.5 7.0 6.5 6.0 5.5 5.0 4.5 4.0 3.5 3.0 2.5 2.0 1.5 1.0 0.5 0.0
f1 (ppm)

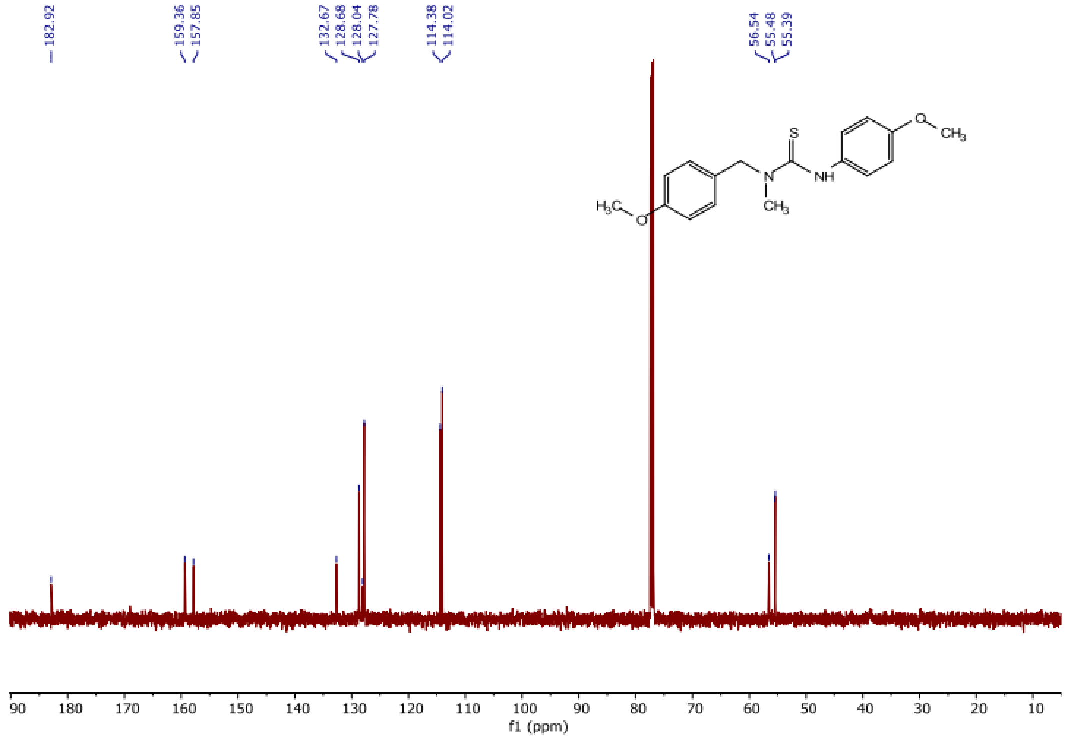

182.92
159.36 157.85
132.67 128.68 128.04 127.78
114.38 114.02
56.54 55.48 55.39
90 180 170 160 150 140 130 120 110 100 90 80 70 60 50 40 30 20 10
f1 (ppm)

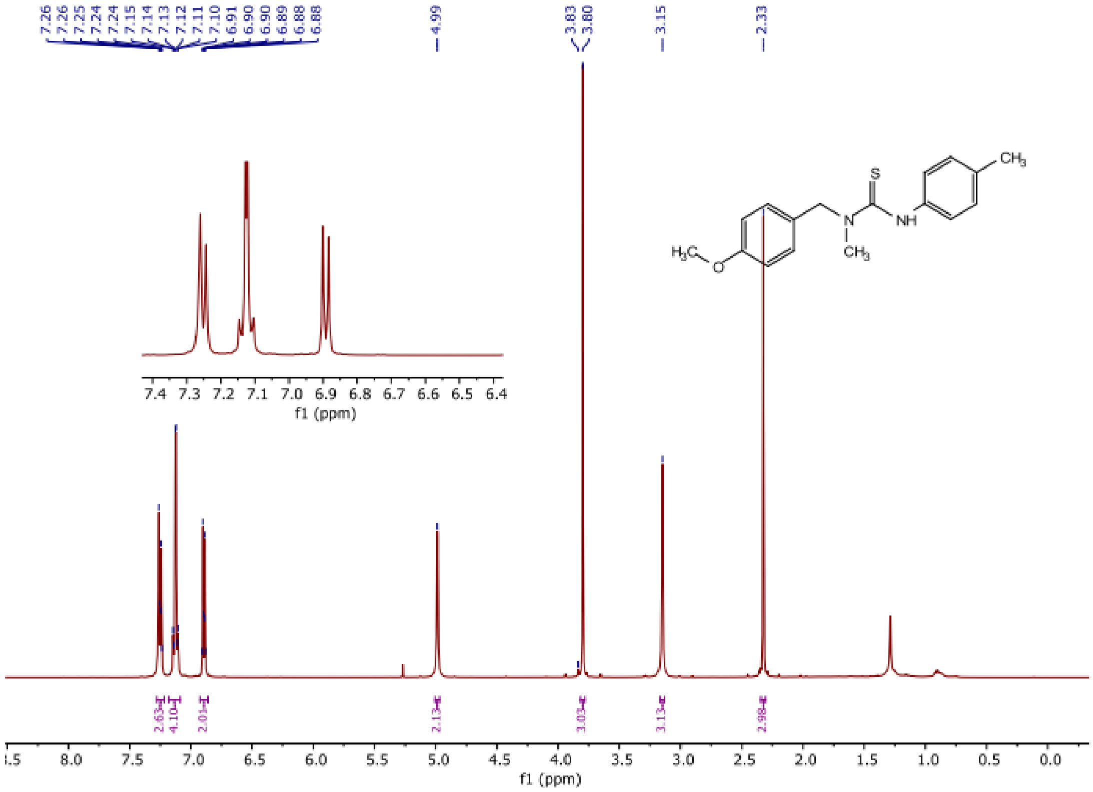

7.26
7.26
7.25
7.24
7.24
7.15
7.14
7.13
7.12
7.11
7.10
6.91
6.90
6.90
6.89
6.88
6.88
4.99
3.83
3.80
3.15
2.33
H_3C
O
N
S
NH
CH_3
CH_3
2.63
4.10
2.01
2.13
3.03
3.13
2.98
f1 (ppm)

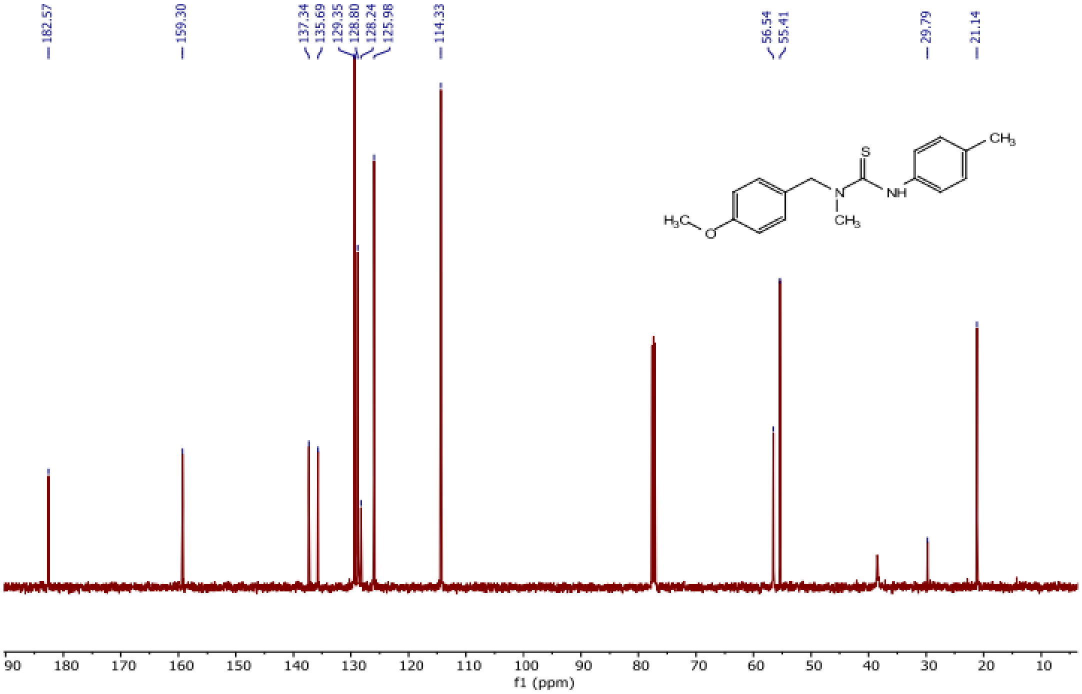

182.57
159.30
137.34
135.69
129.35
128.80
128.24
125.98
114.33
56.54
55.41
29.79
21.14
H_3C
O
N
S
NH
CH_3
CH_3
f1 (ppm)

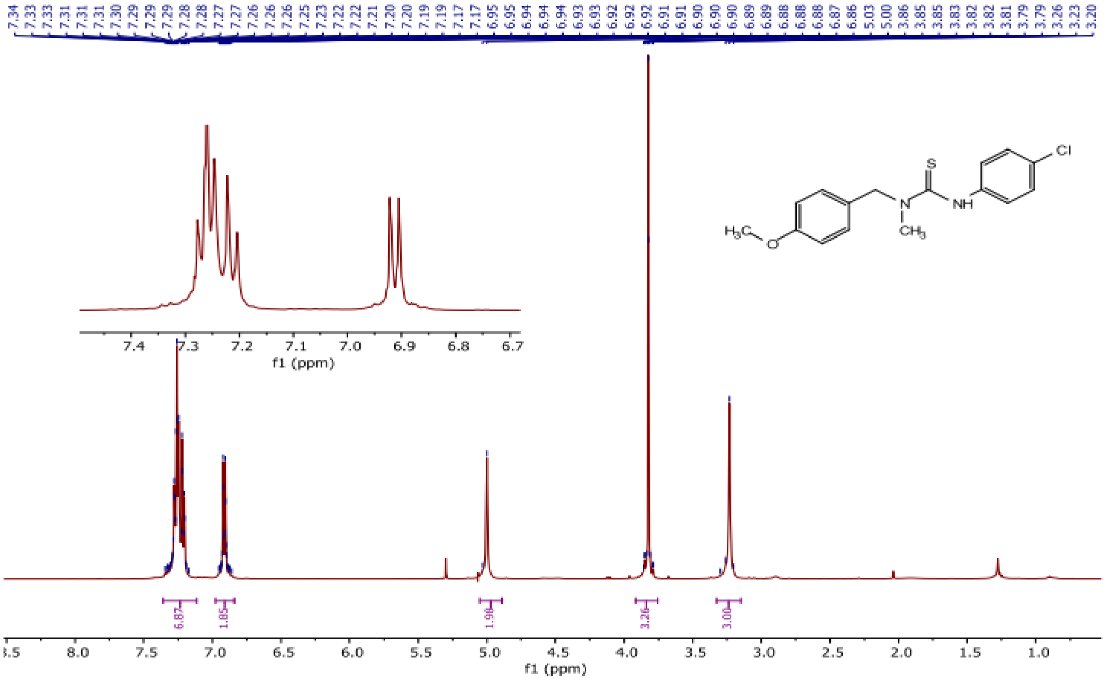

7.34 7.33 7.33 7.31 7.31 7.30 7.29 7.29 7.29 7.28 7.27 7.27 7.26 7.26 7.26 7.25 7.23 7.22 7.22 7.21 7.20 7.20 7.19 7.19 7.17 7.17 6.95 6.95 6.94 6.94 6.94 6.93 6.93 6.92 6.92 6.92 6.91 6.91 6.90 6.90 6.90 6.89 6.89 6.88 6.88 6.88 6.87 6.86 5.03 5.00 3.86 3.85 3.85 3.83 3.82 3.82 3.81 3.79 3.26 3.23 3.20
S
Cl
NH
N
CH3
H3C
O
f1 (ppm)
6.87
1.85
1.98
3.26
3.00
f1 (ppm)

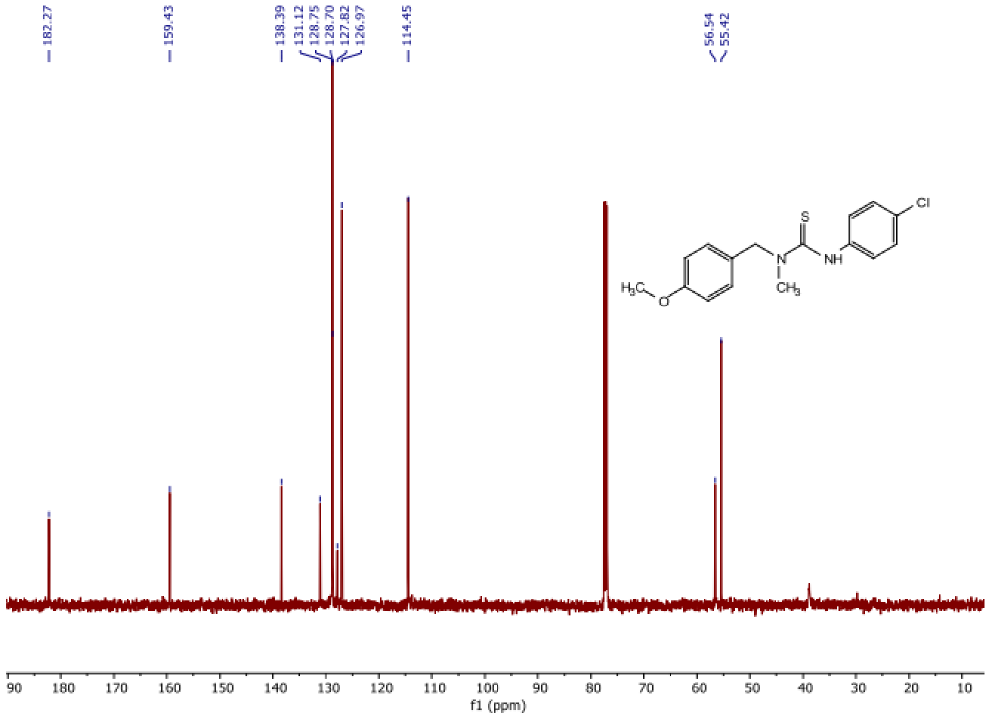

182.27
159.43
138.39
131.12
128.75
128.70
127.82
126.97
114.45
56.54
55.42
S
Cl
NH
N
CH3
H3C
O
f1 (ppm)

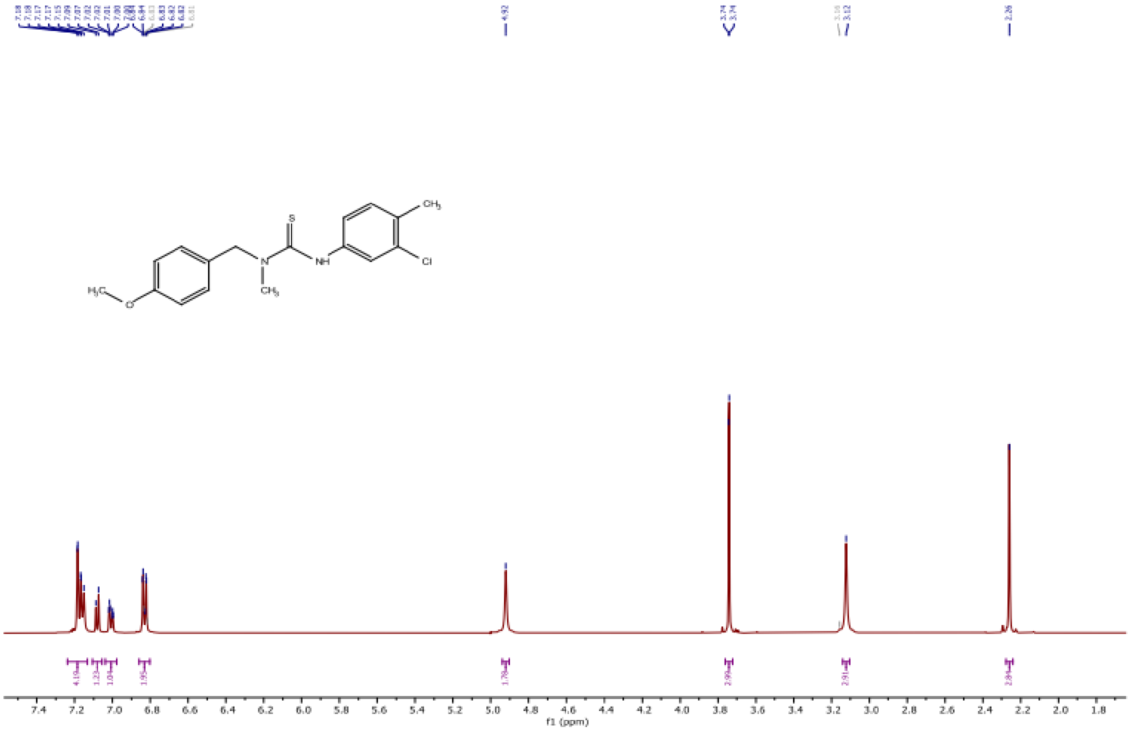
f1 (ppm)

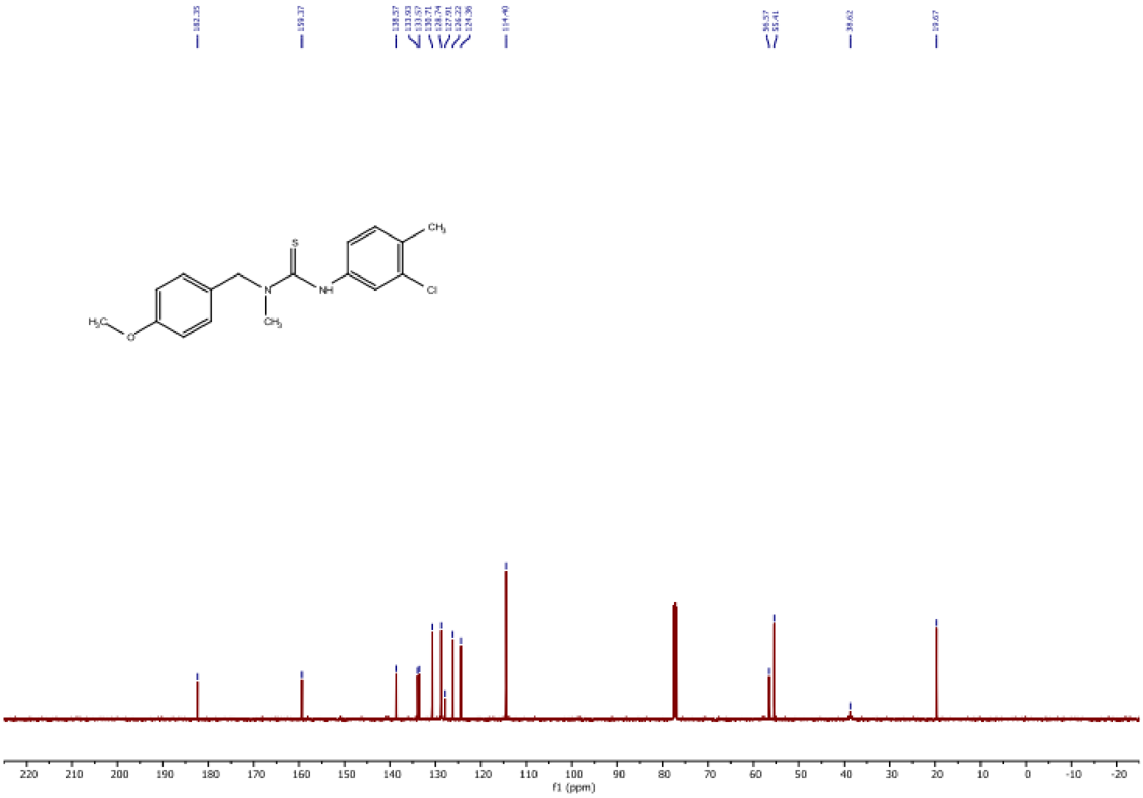
f1 (ppm)

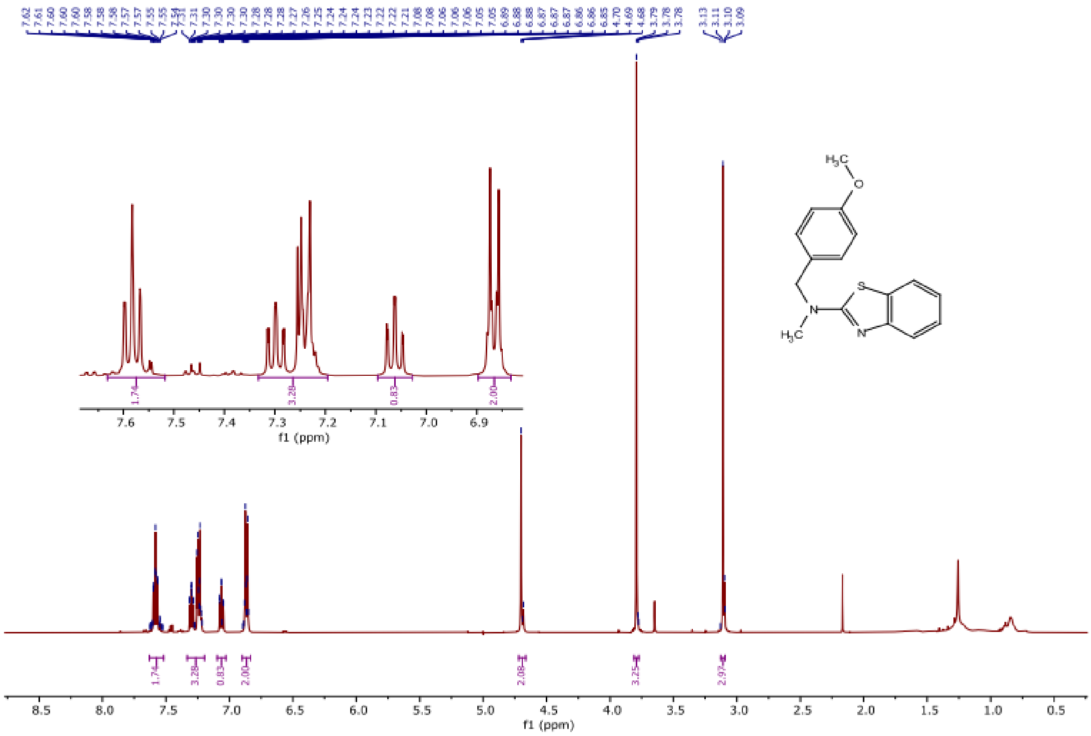

7.62 7.61 7.60 7.60 7.60 7.58 7.58 7.57 7.55 7.55 7.54 7.31 7.30 7.30 7.30 7.30 7.28 7.28 7.28 7.27 7.26 7.25 7.24 7.24 7.24 7.23 7.22 7.22 7.21 7.08 7.08 7.06 7.06 7.06 7.05 7.05 6.89 6.88 6.88 6.87 6.87 6.87 6.86 6.86 6.85 4.70 4.69 4.68 3.79 3.78 3.78
3.13 3.11 3.10 3.09
1.74
3.28
0.83
2.00
2.08
3.25
2.97
f1 (ppm)
H3C
O
S
N
N
H3C

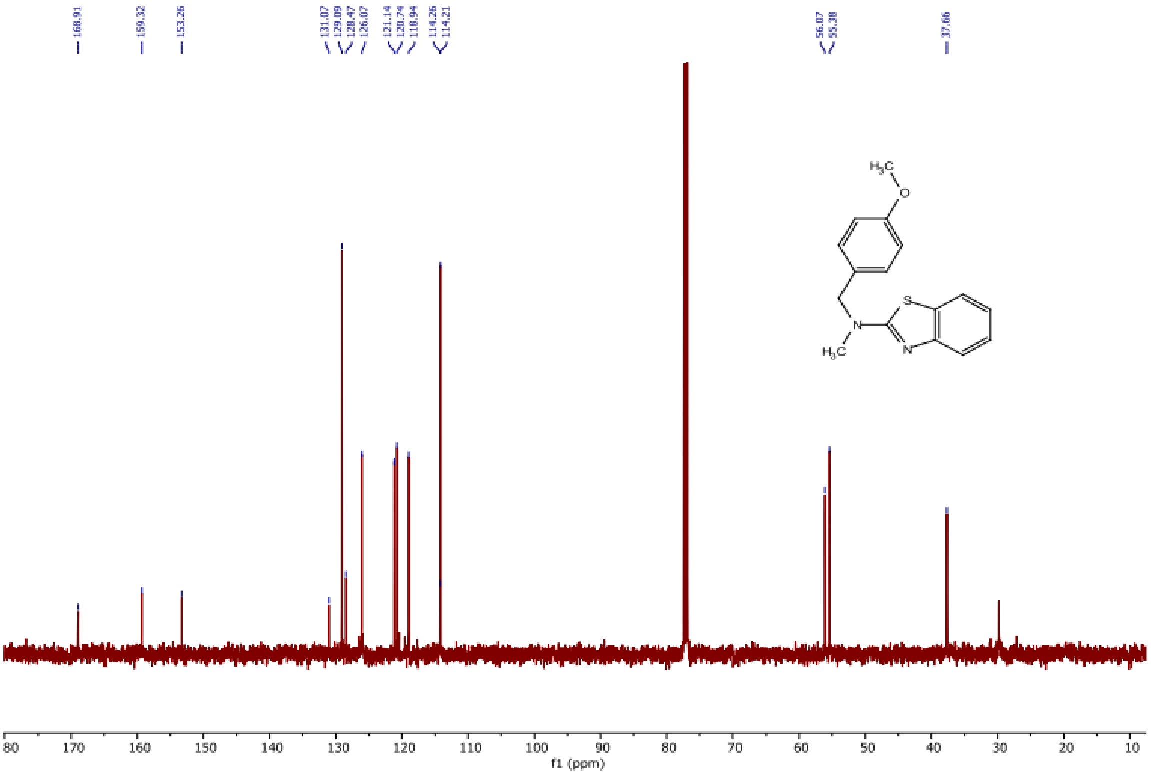

168.91
159.32
153.26
131.07 129.09 128.47 126.07
121.14 120.74 118.94
114.26 114.21
56.07 55.38
37.66
f1 (ppm)
H3C
O
S
N
N
H3C

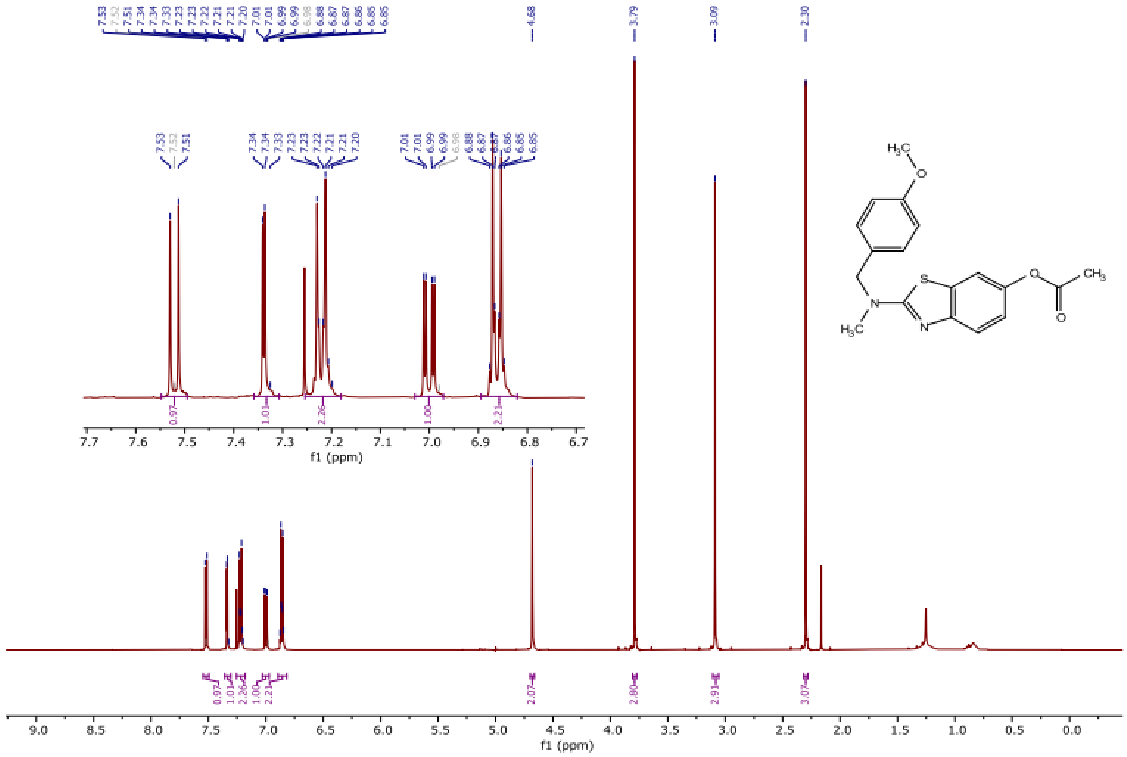
7.53
7.52
7.51
7.34
7.34
7.33
7.23
7.23
7.22
7.21
7.21
7.20
7.01
7.01
6.99
6.99
6.98
6.88
6.87
6.87
6.86
6.85
6.85
4.68
3.79
3.09
2.30
0.97
1.01
2.26
1.00
2.21
2.07
2.80
2.91
3.07
f1 (ppm)
H3C
O
S
N
CH3
O
N
H3C
O

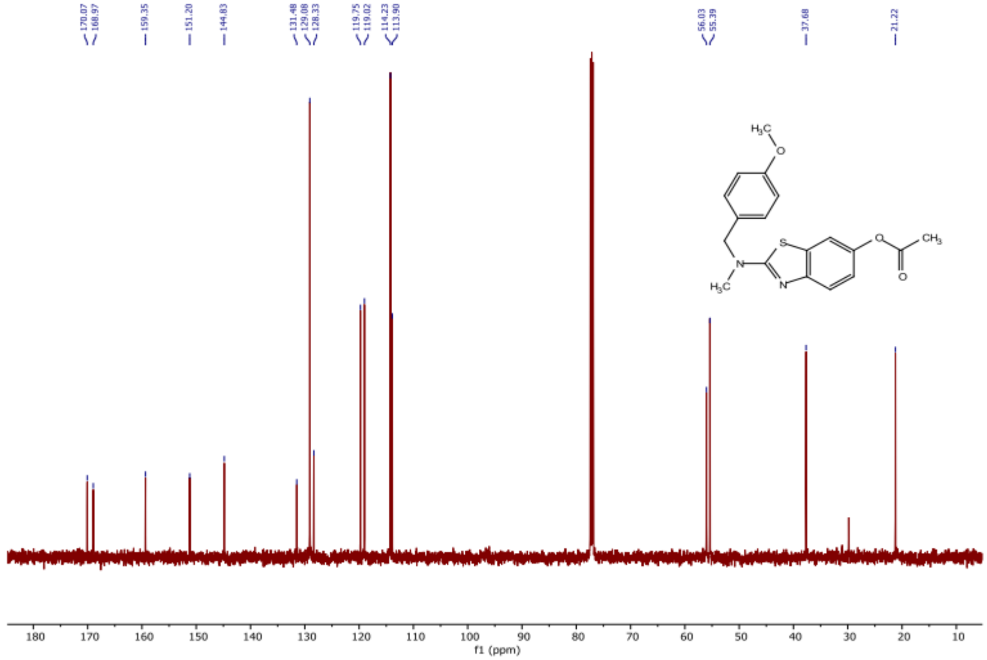
170.07
168.97
159.35
151.20
144.83
131.48
129.08
128.33
119.75
119.02
114.23
113.90
56.03
55.39
37.68
21.22
f1 (ppm)
H3C
O
S
N
CH3
O
N
H3C
O

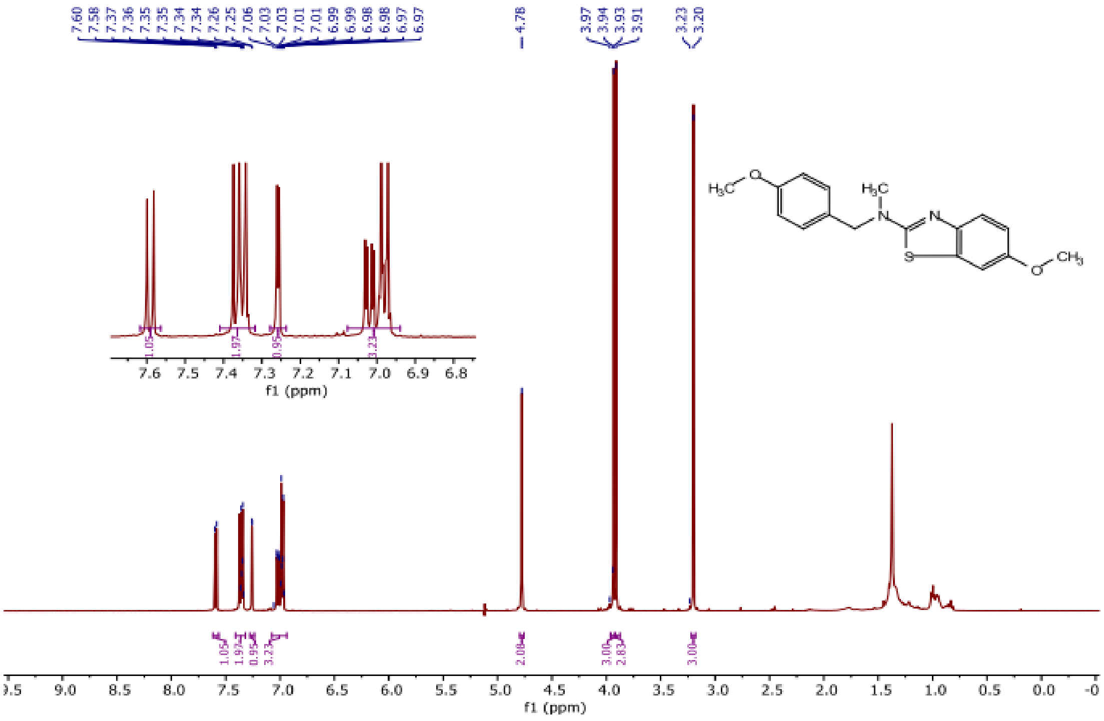
7.60
7.58
7.37
7.36
7.35
7.35
7.34
7.34
7.26
7.25
7.06
7.03
7.03
7.01
7.01
6.99
6.99
6.98
6.98
6.98
6.97
4.78
3.97
3.94
3.93
3.91
3.23
3.20
1.05
1.97
0.95
3.23
f1 (ppm)
2.08
3.00
2.83
3.00
f1 (ppm)

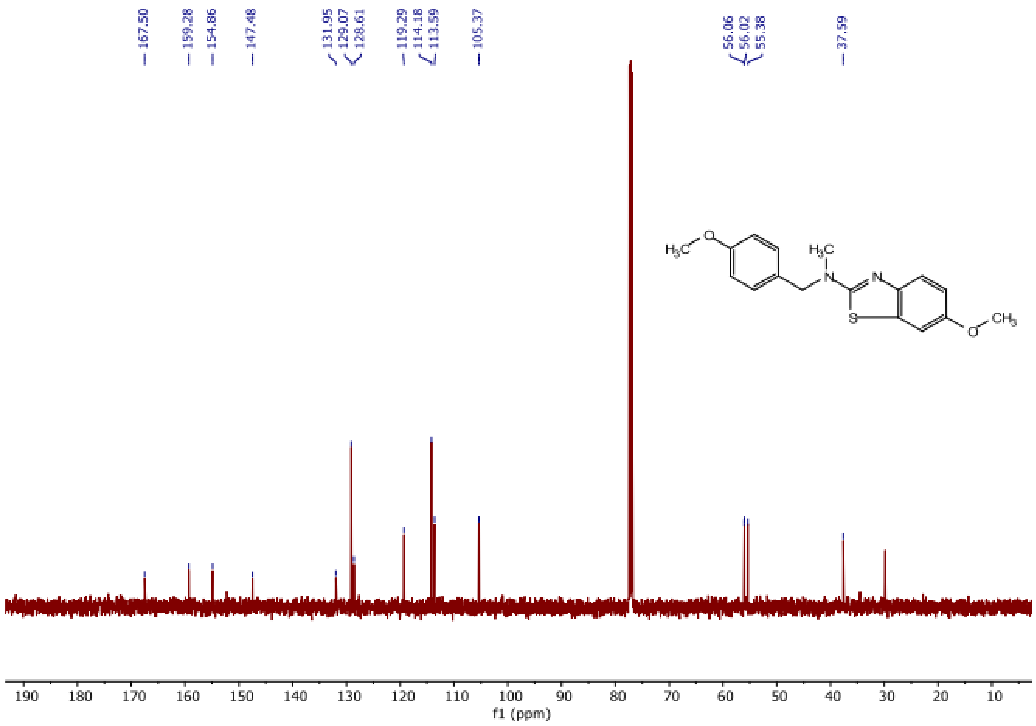
167.50
159.28
154.86
147.48
131.95
129.07
128.61
119.29
114.18
113.59
105.37
56.06
56.02
55.38
37.59
f1 (ppm)

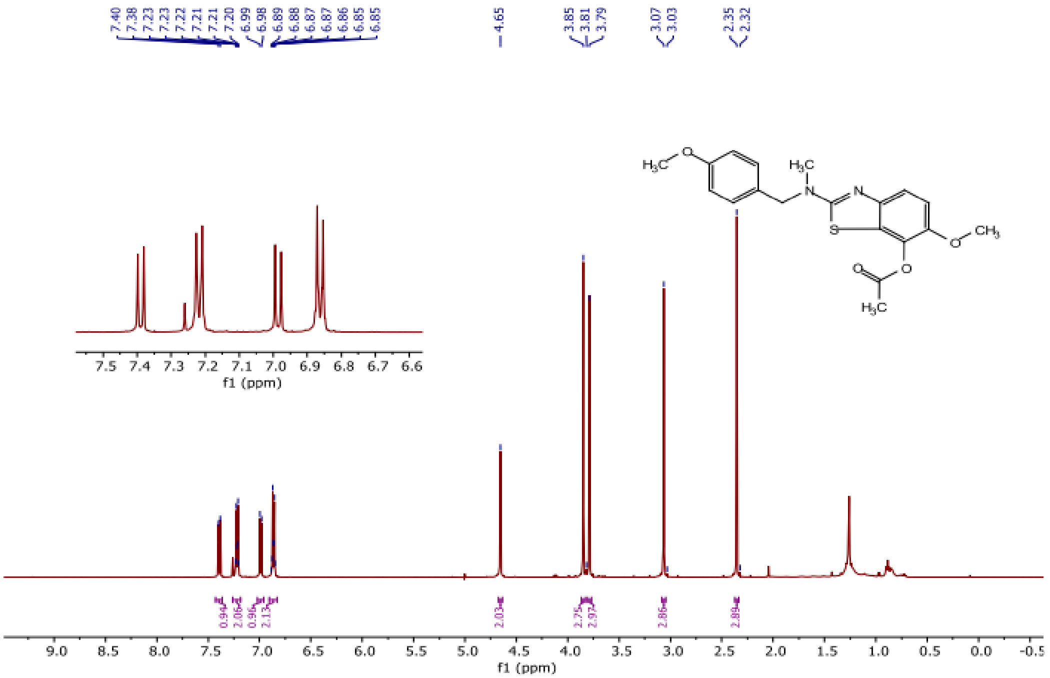
7.40
7.38
7.23
7.23
7.22
7.21
7.21
7.20
6.99
6.98
6.89
6.88
6.87
6.87
6.86
6.85
6.85
4.65
3.85
3.81
3.79
3.07
3.03
2.35
2.32
f1 (ppm)
0.94
2.06
0.96
2.13
2.03
2.25
2.97
2.86
2.89

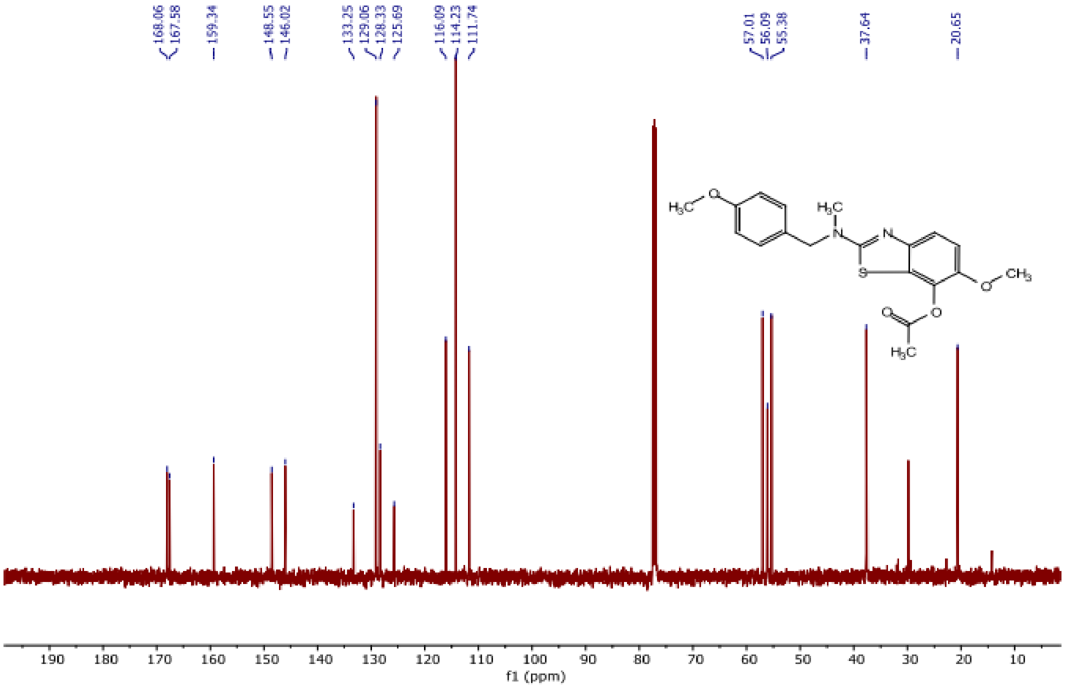
168.06
167.58
159.34
148.55
146.02
133.25
129.06
128.33
125.69
116.09
114.23
111.74
57.01
56.09
55.38
37.64
20.65
f1 (ppm)

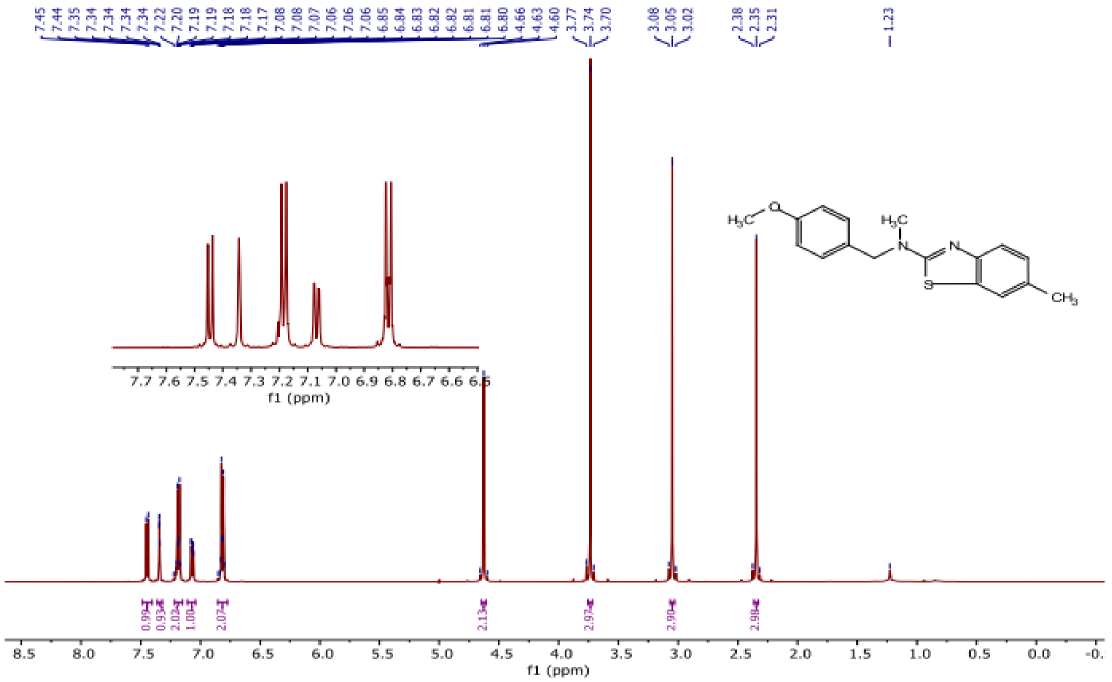

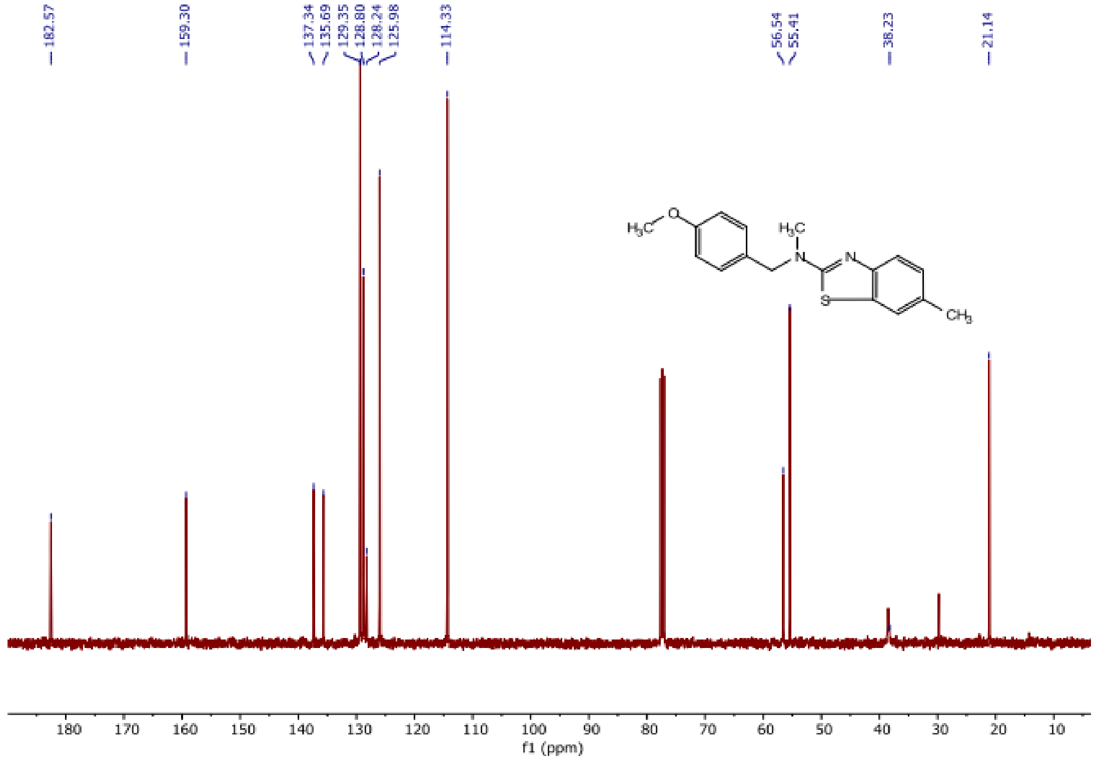

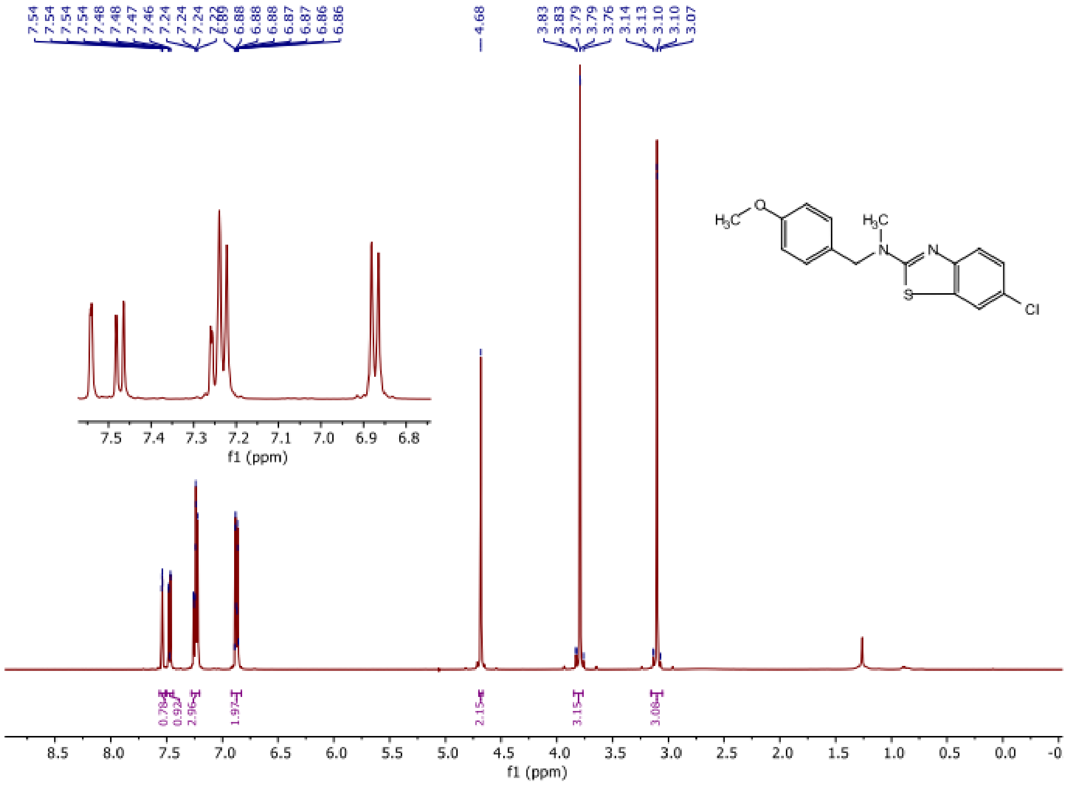

7.54 7.54 7.54 7.54 7.48 7.48 7.47 7.46 7.24 7.24 7.24 7.22 6.89 6.88 6.88 6.88 6.87 6.86 6.86
4.68
3.83 3.83 3.79 3.79 3.76 3.14 3.13 3.10 3.10 3.07
H3C O
H3C N
N
S
Cl
f1 (ppm)
0.78 0.92 2.96 1.97 2.15 3.15 3.08
f1 (ppm)

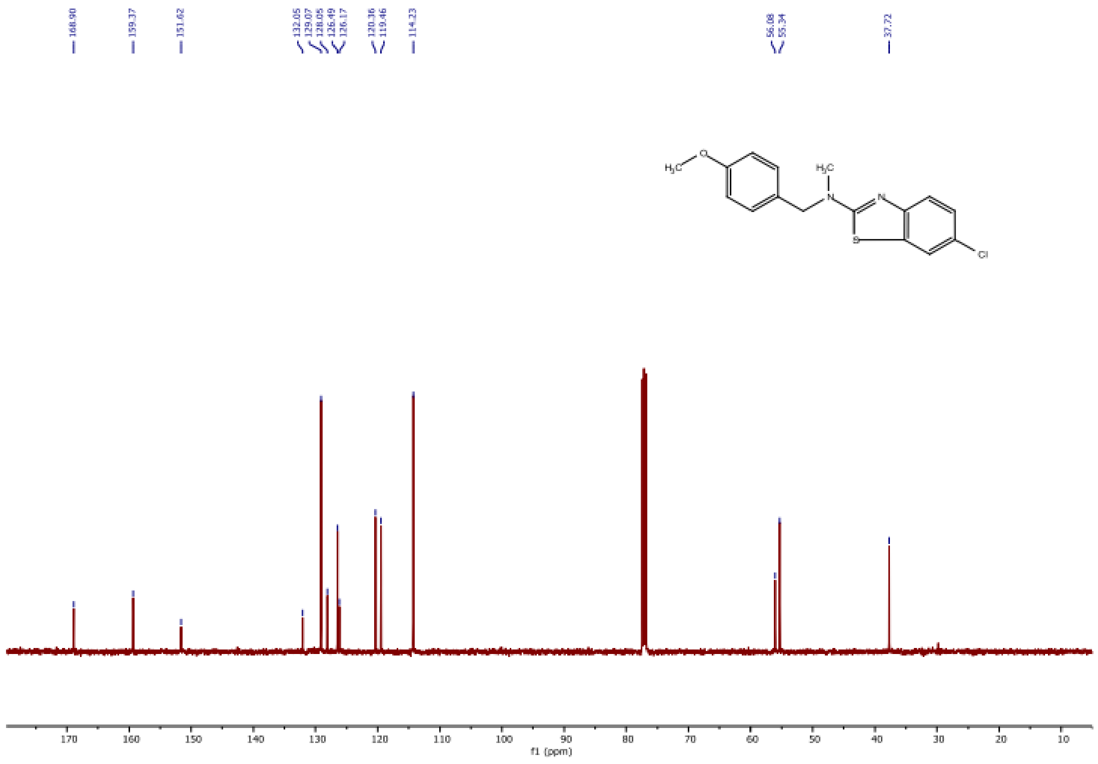

168.90
159.37
151.62
132.05 129.07 128.05 126.49 126.17
120.36 119.46
114.23
56.08 55.34
37.72
H3C O
H3C N
N
S
Cl
f1 (ppm)

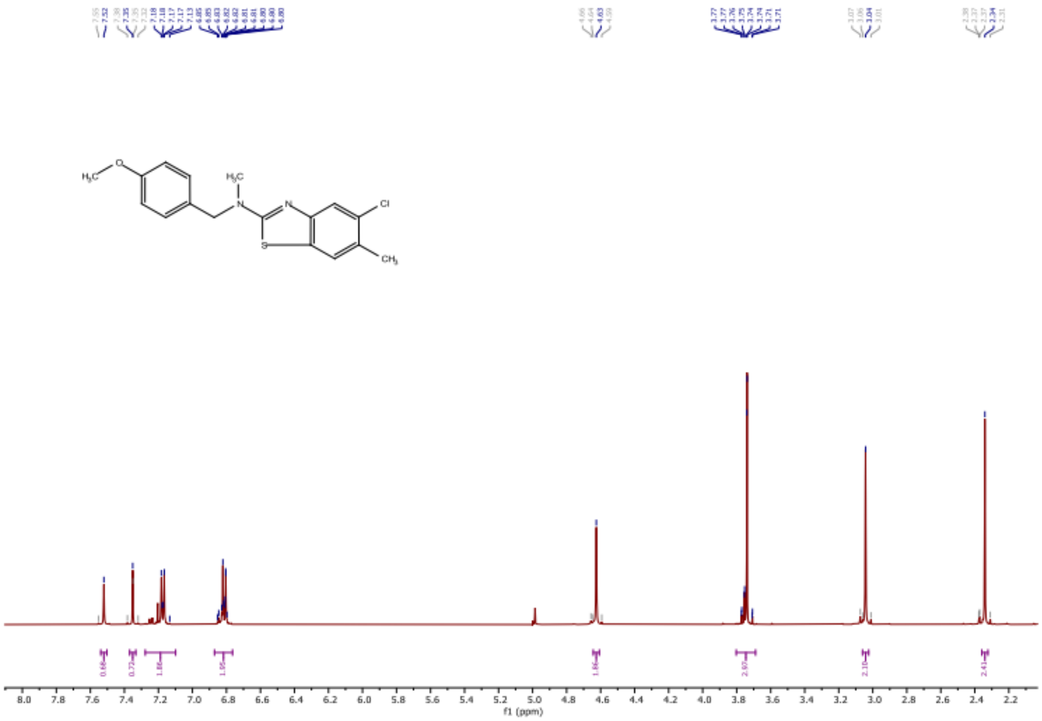

f1 (ppm)

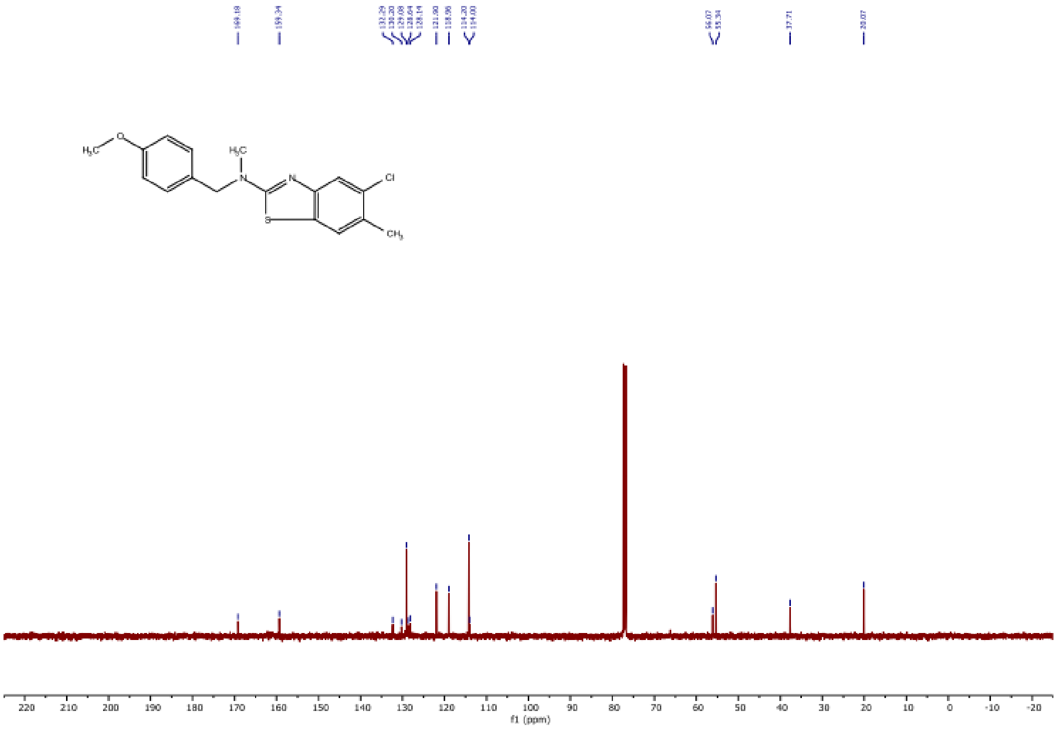

f1 (ppm)
